# Association between uveitis onset and economic development in mainland China

**DOI:** 10.1186/s12889-023-16591-x

**Published:** 2023-09-04

**Authors:** Handan Tan, Xiaojie Feng, Peizeng Yang

**Affiliations:** The First Affiliated Hospital of Chongqing Medical University, Chongqing Key Lab of Ophthalmology, Chongqing Eye Institute, Chongqing Branch of National Clinical Research Center for Ocular Diseases, Chongqing, People’s Republic of China

**Keywords:** Uveitis, Gross domestic product, Disease burden

## Abstract

**Background:**

Economic prosperity has fostered numerous changes that may translate into better or worse outcomes across all domains of health. This study aims to explore the associations of economic development with uveitis onset in mainland China.

**Methods:**

We used Poisson regression with generalized estimated equations to quantify the associations of per capita gross domestic product (GDP) with uveitis onset in 31 provinces of mainland China from 2006 to 2017. We further estimated the effects mediated by economic growth on the temperature-uveitis and PM2.5-uveitis associations established in our previous studies.

**Results:**

A total of 12,721 uveitis patients from 31 provinces of mainland China were studied. Overall, every 10,000 Chinese yuan ($ 1491.278, 2006–2017) increase in per capita GDP, with no weighted value or weighted by population, corresponded to 1.85% (95% confidence interval, 1.19–2.52%) and 1.43% (95% confidence interval, 0.37–2.51%) lnRR decrease in the uveitis onsets. Stratified analysis showed this negative association between per capita GDP and uveitis onset, only existed in male patients (*P* < .001), individuals aged 20–50 years (*P* < 0 .05), non-infectious uveitis, uveitis with systemic disease, and Bechet’s disease (all *P* < 0 .05). Moreover, the increased per capita GDP, if above the national level, could reinforce both temperature-uveitis and PM2.5-uveitis association (both *P* < 0.001).

**Conclusions:**

The findings suggest that economic development is negatively associated with uveitis onset. However, it may facilitate the uveitis onset mediated by both increased temperature and PM2.5 exposure if the per capita GDP is above national level.

**Supplementary Information:**

The online version contains supplementary material available at 10.1186/s12889-023-16591-x.

## Background

Uveitis refers to a group inflammation affecting the uvea, retina, retinal blood vessels as well as vitreous body [1, 2]. There are numerous uveitis entities, including infectious, non-infectious, and traumatic inflammation [3, 4]. It is one of the common causes of blindness, accounting for 15% of visual impairments in the world [5, 6]. Uveitis and its associated complications account for up to 25% cases of blindness in developing countries [5, 7]. It commonly occurs in the working-age population [8]. This often has a profound impact on people’s quality of life and might have a great socioeconomic implication.

It has been shown that socioeconomic status and economic prosperity has fostered numerous changes which may translate into better or worse outcomes across all domains of health. Previous studies have report that lower socioeconomic status is associated with a higher incidence rate of some inflammatory and autoimmune diseases, such as rheumatoid arthritis, Graves' disease, and systemic lupus erythematosus [9–11]. Uveitis may be associated with systemic inflammatory and autoimmune disorders [12]. It has been suggested that socioeconomic disparities may play a significant role in the disease and emphasized the need for targeted interventions to reduce the burden of the disease. Recent studies have shown that gross domestic product (GDP) is significantly associated with health status of the populations [13, 14]. Some studies have reported that GDP is also significantly correlated with the clinical status of immunoinflammatory diseases [15, 16] and infectious and immune diseases are more common in the low GDP population [17–19].

However, to the best of our knowledge, there is no analysis to investigate the association between the onset of uveitis and economic development in China. China, one of the world’s largest populations, has experienced unprecedented economic growth in the past decades. Also, uveitis in China may be the largest component of the emerging uveitis epidemic in the world. If economic development has caused even modest changes in the onset of uveitis, this may be a significant challenge to global health. Therefore, it is important to determine whether uveitis is associated with economic growth.

In this study, we sought to explore the association between the onset of uveitis and economic development over time and to describe the trend of the uveitis onset in the context of per capita GDP during 2006 to 2017.

## Methods

### Data

This study was based on the largest documentary management system and medical database for uveitis patients across mainland China including 31 provinces [20]. These data have been described in detail in previous publications [20–22]. Patients with unknown onset times were excluded. A total number of 12,721 cases was enrolled in our study and the time span was from January 2006 to December 2017. We matched the data for the time (year) and location (province) of uveitis onset to data for the annual provincial per capita GDP. Our study took per capita GDP as an indicator of economic development. The GDP and per capita GDP were sourced from the China Statistical Yearbook (2006–2017). We also obtained the data of provincial populations from the China Statistical Yearbook to ensure that all regression analyses were weighted by population. Meteorological data and air pollution data described in previous publications [21, 22] were also used to analyzed the interaction either between per capita GDP and increased temperatures or between it and particulate matters less than 2.5 μm (PM2.5) exposure in the development of the uveitis.

### Statistical analysis

We used a choropleth map (ArcGIS Version 10 software) to visually examine the spatial variations in the proportion of uveitis (the provincial uveitis case numbers divided by the total number of cases) in the 31 provinces across mainland China. All participants from 31 provinces were nested into, 1) the northern provinces: Beijing, Hebei, Shanxi, Tianjin, and Inner Mongolia; 2) the southern provinces: Guangdong, Guangxi, and Hainan; 3) the eastern provinces: Anhui, Fujian, Jiangsu, Jiangxi, Shanghai, Shandong, and Zhejiang; 4) the northwestern provinces: Gansu, Ningxia, Qinghai, Shaanxi, and Xinjiang; 5) the northeastern provinces: Heilongjiang, Jilin, and Liaoning; 6) the southwestern provinces: Chongqing, Sichuan, Guizhou, Tibet, and Yunnan; and 7) the central provinces: Henan, Hubei, and Hunan.

We used Poisson regression with generalized estimated equation (Poisson-GEE) to quantify the associations of per capita GDP with risk ratio (RR) and 95% percent confidence (CI) of uveitis onset. We mainly estimated the β coefficient (effect size in the equation), to provide lnRR of uveitis onset associated with a per 10,000 Chinese yuan ($ 1491.278, the average exchange rate was 6.706 between 2006 and 2017) increase in GDP per capita. Stratified analyses were performed by sex (male and female), age (aged 20–50 years and others) [5], uveitis entities (uveitis alone or associated with systemic diseases, infectious or noninfectious uveitis, idiopathic uveitis and two common uveitis entities: Vogt-Koyanagi-Harada disease (VKH) and Bechet’s disease (BD)), and survey year (2006–2017). All regression analyses were weighted and unweighted by province population, respectively. We used R version 3.5.0 to conduct all these analyses. Two-sided *p* < 0.05 is considered statistically significant.

To further estimate the economic activity mediated effects on the temperature-uveitis and the PM2.5-uveitis association, we interacted these two exposures with per capita GDP dummy variables. The modeling methods have been described in detail in previous studies [21, 22].

## Results

A total of 12,721 uveitis patients with a mean (standard deviation) age of 36.2 (16.2) years were included in this analysis. Male and female accounted for 52.6% and 47.4%, respectively. The sample sizes ranged from 474 to 12,247 in each sex, age, and type subgroups. The most common age of onset of uveitis was 20–50 years old [5], accounting for 64.7% in this study. The characteristics of enrolled patients were described in detail elsewhere [21, 22] (Table 1, Table S1 in Supplementary Appendix and Fig. 3).

**Table 1:** Characteristics of the included patients

Characteristic	Value	
	**No**	**%**
**Group of age at onset**		
20–50 years	8229	64.7
Others	4492	35.3
**Sex**		
Male	6695	52.6
Female	6026	47.4
**Types of uveitis**		
Idiopathic uveitis	5895	46.3
Vogt-Koyanagi-Harada disease	1663	13.1
Behçet’s disease	1260	9.9
Uveitis alone	8615	67.7
Uveitis with systemic diseases	4106	32.3
Infectious uveitis	474	3.7
Non-infectious uveitis	12,247	96.3
Total	12,721	100

### Trend in the onset of uveitis and in per capita GDP

**Fig. 1:**
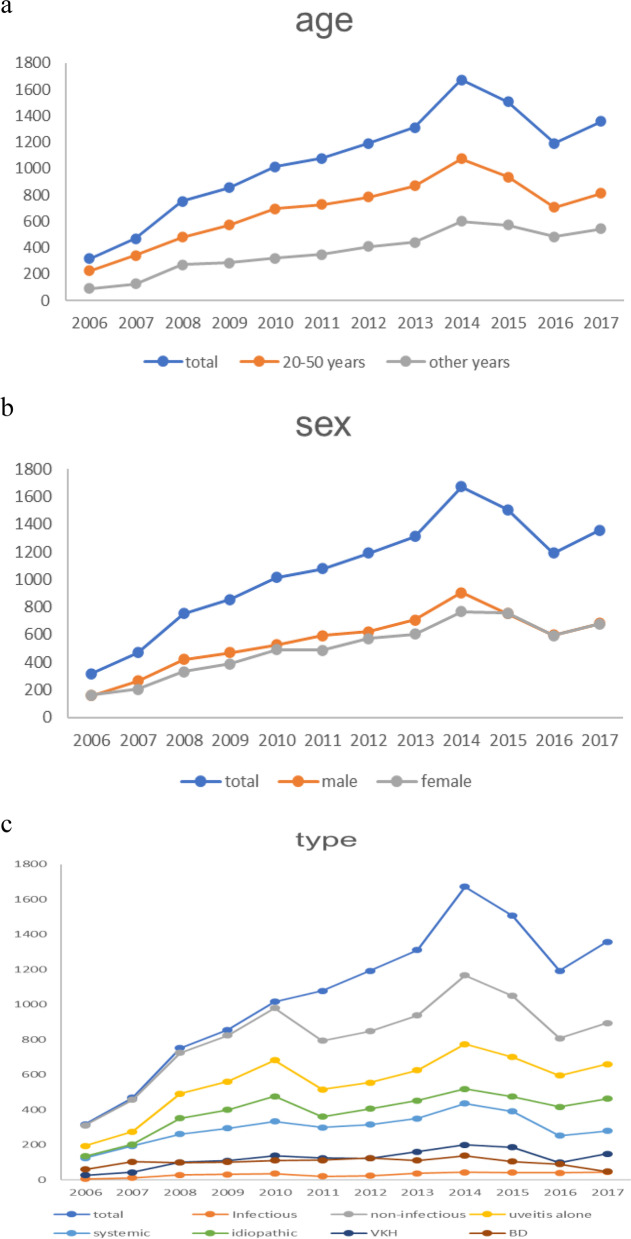
Tends of the uveitis onset from 2006 to 2017, stratified by sex, age and type. **a** Age group. **b** Sex subgroup. **c** Type subgroup. X axis: year. Y axis: Number of cases

**Fig. 2:**
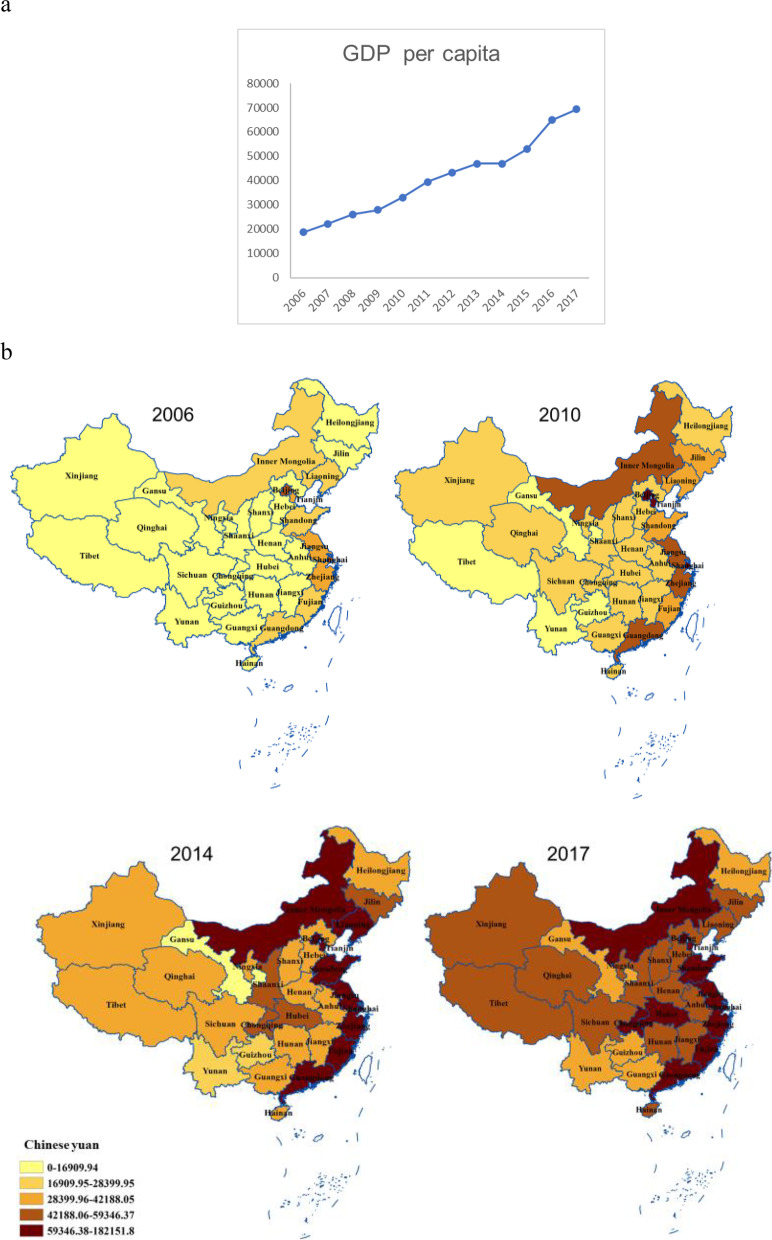
Trend and geographical distribution of economic development in mainland China from 2006 to 2017. **a** Trend of the gross domestic product (GDP) per capita from 2006 to 2017. X axis: year. Y axis: Chinese yuan. **b** Geographical distribution of the GDP per capita. We used quintiles to define map strata

**Fig. 3:**
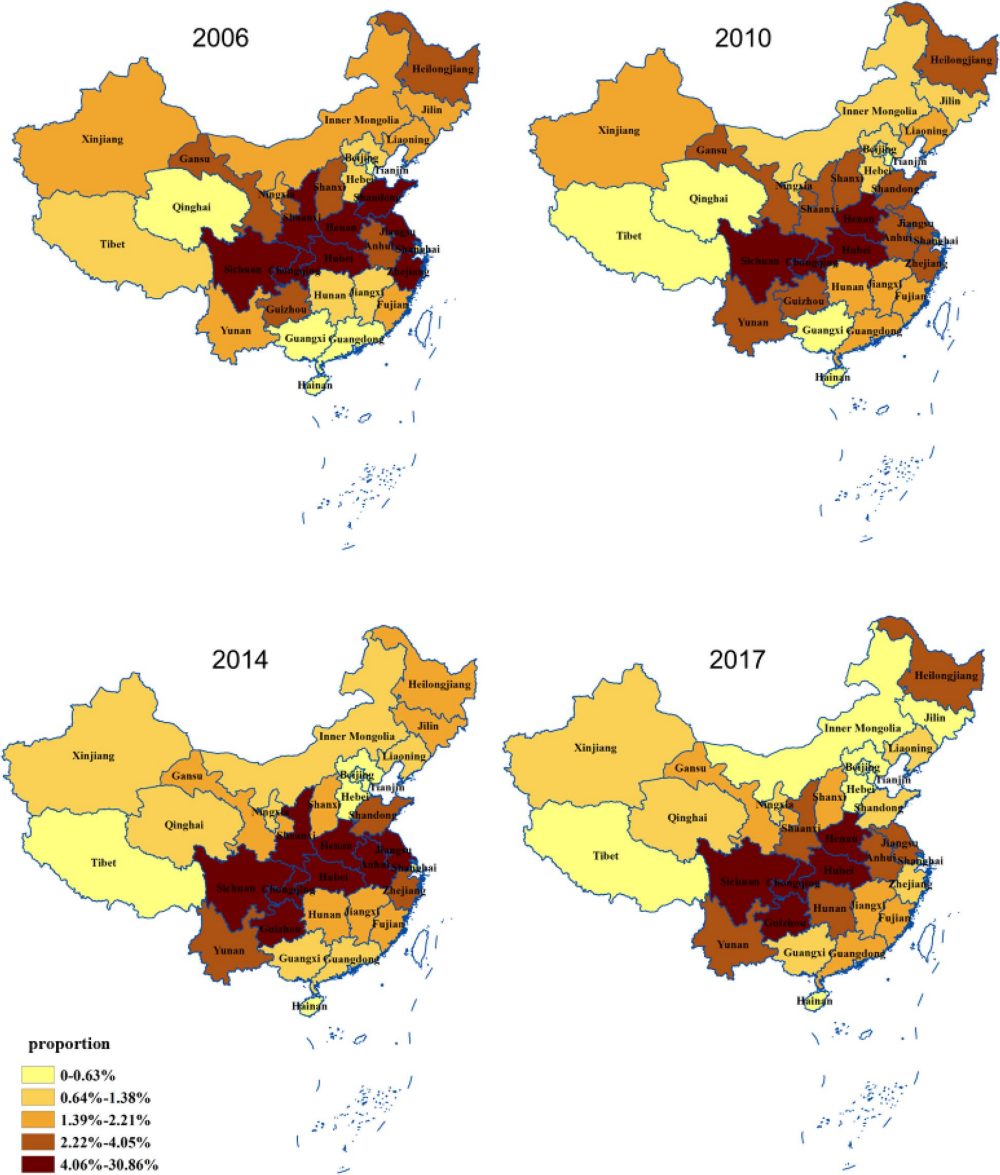
Geographical distribution of uveitis onset in mainland China from 2006 to 2017. The proportion of patients with uveitis in mainland China by provinces. We used quintiles to define map strata

An increased trend of the onset of uveitis was observed during the investigation, both in total and stratified by sex, age and uveitis type (Fig. 1). The per capita GDP significantly increased by more than 3.5 times from 18,782 to 69,365 Chinese yuan in mainland China from 2006 to 2017 (Fig. 2a). Although these choropleth maps showed a marked spatial variation in the onset of uveitis across mainland China, there was no significant change in uveitis proportion in each province from 2006 to 2017 (Fig. 3). However, there was significant difference in per capita GDP in each province between 2006 and 2017 (Fig. 2b).

### Association between the onset of uveitis and economic development

**Fig. 4:**
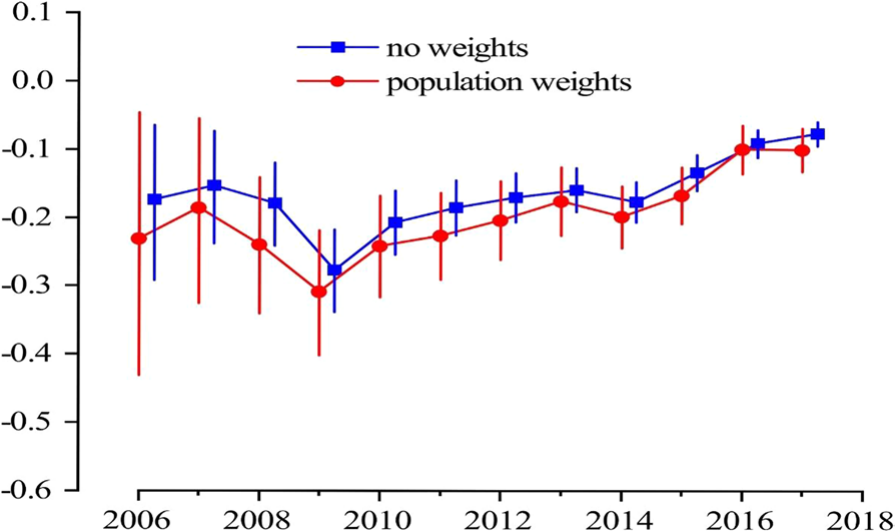
Associations of economic development with uveitis onset over time. X axis: year. Y axis: effect size (β) and 95% confidence intervals (CI)

**Fig. 5:**
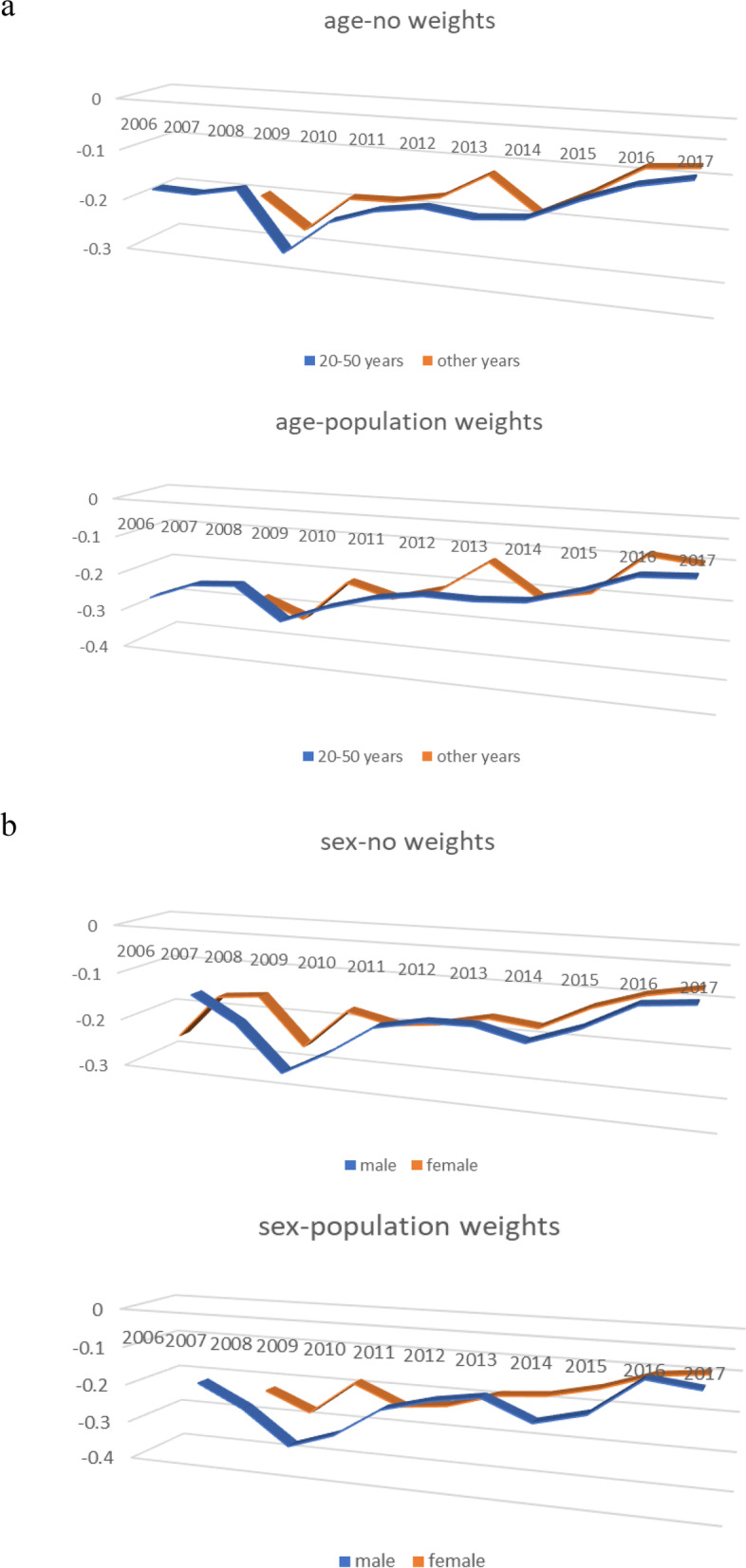
Effects of economic development on age-adjusted and sex-adjusted onset of uveitis over time. **a** Age-adjusted uveitis onset without or with weighted value. **b** sex-adjusted uveitis onset without or with weighted value. X axis: year. Y axis: effect size (β)

According to the regression model weighted by or without by population, we found that every 10,000 Chinese yuan ($ 1491.278, 2006–2017) increase in per capita GDP was associated with 1.85% (95% confidence interval, 1.19–2.52%) and 1.43% (95% confidence interval, 0.37–2.51%) lnRR decrease in the onset of uveitis, respectively (Table S2 in Supplementary Appendix). Stratified by age, we found a significantly negative association of per capita GDP and uveitis onset only in patients aged 20–50 years (no weighted value: β, -2.81%; *P* < 0.001; weighted by population: β, -2.52%; *P* < 0.001). There was a negative association of per capita GDP with uveitis onset both in males (β, -2.40%; *P* < 0.001) and females (β, -1.26%; *P* < 0.001) under unweighted condition. However, this association was only present in males (β, -2.01%; *P* < 0.01) if weighted by population. We further used the Poisson-GEE model to evaluate this association over time. The result showed that the negative associations of economic development with the onset of uveitis was getting weaker no matter with or without weighting over time (Fig. 4). A similar trend was also observed when stratified by age and sex (Fig. 5). A study with stratification by uveitis types, showed that the aforementioned negative association was present in BD, non-infectious uveitis, and uveitis with systemic disease. The strongest negative association was found in BD subgroup (Table S2 in Supplementary Appendix).

### Per capita GDP-mediated effect on the PM2.5-uveitis and the temperature-uveitis association

Our previous study revealed that particulate matter with diameters < 2.5 mm (PM2.5) and temperature were positively associated with an increased in uveitis onset. We further used a fixed–effect model containing an interaction factor to test the influence of per capita GDP on the PM2.5-uveitis association or the temperature-uveitis association [21, 22]. The result showed that PM2.5 exposure could increase uveitis onset in all the areas when climate control variables were not included (Table S3 in Supplementary Appendix). When analyzed with climate control variables, we found a positive correlation between PM2.5 increases and the onset of uveitis only in the areas where per capita GDP was above the national level (Table S3 in Supplementary Appendix). We also found a similar effect of per capita GDP on the temperature-uveitis association (Figure S1 in Supplementary Appendix).

## Discussion

In this study, we investigated the association between economic development and uveitis onset and the effects mediated by economic growth on both the temperature-uveitis association and the PM2.5-uveitis association. The result showed that economic development itself had a negative association with the onset of uveitis either across all the samples or stratified by age, sex and uveitis type. Overall, the negative associations of economic development with the onset of uveitis were getting lower over time. However, the economic development could facilitate the uveitis onset mediated by both increased temperatures and PM2.5 exposure if the per capita GDP is above national level.

A growing body of literature has revealed the associations of economic growth with other immune and inflammatory diseases [16, 23, 24]. Previous studies have shown that the burden of arthritis appears substantially greater in low GDP area than in high GDP area among 25 European countries [15]. The use of biologic disease-modifying antirheumatic drugs might improve rheumatoid arthritis in low GDP area [25, 26]. In this study, we addressed this issue using a relatively large database of uveitis patients from mainland China with a span of 12 years. The results showed that an increased per capita GDP was negatively association with uveitis onset across all the samples investigated. Importantly, we found this negative association was only present in the individuals aged 20–50 years, males and the uveitis entities including BD, non-infectious uveitis, uveitis with systemic disease. The reason as to why an increased per capita GDP could decrease the development of uveitis is not fully understood. Based on a biopsychosocial model to complement a biomedical model, a number of psychological and mental abnormalities, such as stress, anxiety, and depression, were usually associated with poor health status in individuals [27–30]. Psychological distress appeared significantly greater in low GDP countries than in high GDP countries [15]. Psychological and mental disorders might lead to a dysregulation of the immune response and a pro-inflammatory state in the body [31, 32]. An increased GDP may lead to a decreased uveitis onset possibly through improvement of people’s psychological and mental status and immune function. Another possibility is that deceased uveitis onset may be due to improvement of people’s hygiene status arising from economic development. Unexpectedly, we failed to find any association between infectious uveitis and economic development. Indeed, infectious diseases, such as tuberculosis, are often associated with low GDP population [17–19]. The reason as to this result presented in this study are not completely understood. One of reasons is that vaccination is broadly given to the individuals in China and immune response against this infection is initiated universally, therefore decreasing the occurrence of this disease [33–35]. More studies are expected to clarify this issue in the future studies. An interesting observation is that the most robustly negative association exits between per capita GDP and BD in our study. It has been reported that there is a gradually decreased incidence of BD in Japan during recent decades [36]. This decreased BD incidence [36, 37] may be largely attributed to the awareness and improvement of people’s hygiene. It is likely that an increased per capita GDP could indirectly decrease the development of BD and other uveitis types in China through improvement of the people’s hygiene status.

One of important results in our study is that the negative influence of per capita GDP on the uveitis onset was not observed in a fixed–effect model analysis using PM2.5 and the climate data. Our previous study showed that PM2.5 and temperature were positively associated with an increased in uveitis onset [21, 22]. Unexpectedly, an increased per capita GDP, if above the national level, could reinforce both the temperature-uveitis association and the PM2.5-uveitis association. The reason for this result is not clearly understood. It has been reported that rapid economic development may contribute to an increased ambient temperature and high PM2.5 exposure [38, 39]. The negative modulation of an increased per capita GDP on uveitis onset may be neutralized by the effect of increased temperature or PM2.5 exposure. This could, at least partially, explain the disappearance of negative effect of per capita GDP on uveitis onset observed in the current study. Another finding in our study is that the negative association of per capita GDP on uveitis onset was getting weaker over time. This result also supports the neutralized effect of increased ambient temperature and PM2.5 exposure on the negative modulation of uveitis onset mediated by per capita GDP.

There are several limitations in our study. There may be a selection bias regarding the enrollment of patients, because the study was carried out on patients treated at one referral center and richer patients were easily being referred to our uveitis center. As a single center study, the representativeness is relatively weak despite the relatively large sample size in our study. Secondly, we cannot establish an exact causality between the onset of uveitis and increased per capita GDP due to the fact that there may be big difference in economic levels among different areas within the same province. More prospective studies with strict controls are needed to address this issue. Thirdly, there are the numerous types of uveitis [3, 40]. Our study was unable to determine the exact impact of per capita GDP on each type of uveitis. Finally, and importantly, the mechanisms under the effect of per capita GDP on uveitis should be investigated in future studies.

In conclusion, our study suggests that the economic growth, by itself or through some main environmental factors, is associated with the onset of uveitis. These comprehensive patterns may also have implications for other developing countries experiencing rapid economic development and for studying the development of prevention and treatment strategy in uveitis.

### Supplementary Information

**Additional file 1:** **Fig. S1.** GDP-mediated temperature-uveitis relationship. The effect of variation in temperature on the number of uveitis cases when climate models contain an interaction-per capita GDP. The dots are point estimates of the effect of monthly temperature on monthly uveitis cases; the lines are 95% CI. **Table S1.** The proportion of patients with different uveitis subtypes in Mainland China by provinces, 2006-2017. **Table S2.** Associations of uveitis onset with GDP per capita*. **Table S3.** Per capita GDP-mediated 2.5–uveitis relationship.

## Data Availability

All data generated or analysed during this study are included in this published article.
