# Supplementary material for: Association between uveitis onset and economic development in mainland China
Source: BMC Public Health. 2023 Sep 4;23:1711. doi: 10.1186/s12889-023-16591-x (PMC10476315; doi:10.1186/s12889-023-16591-x)
Supplement: Supplementary file 1 — Additional file 1: Fig. S1. GDP-mediated temperature-uveitis relationship. The effect of variation in temperature on the number of uveitis cases when climate models contain an interaction-per capita GDP. The dots are point estimates of the effect of monthly temperature on monthly uveitis cases; the lines are 95% CI. Table S1. The proportion of patients with different uveitis subtypes in Mainland China by provinces, 2006-2017. Table S2. Associations of uveitis onset with GDP per capita*. Table S3. Per capita GDP-mediated 2.5–uveitis relationship. [file 12889_2023_16591_MOESM1_ESM.docx]

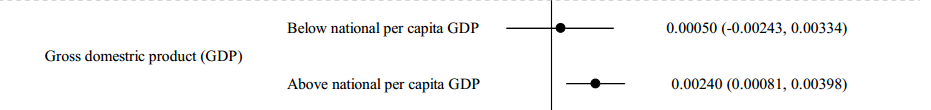


**Figure S1. GDP-mediated temperature-uveitis relationship.** The effect of variation in temperature on the number of uveitis cases when climate models contain an interaction-per capita GDP. The dots are point estimates of the effect of monthly temperature on monthly uveitis cases; the lines are 95% CI.

| Types | Highest quintile | Lowest quintile |
| --- | --- | --- |
| Infectious uveitis | Hainan | Jilin, Inner Mongolia, Guizhou, Shanghai, Gansu, Tianjin, Ningxia |
| Non-infectious uveitis | Gansu, Ningxia, Shangha, Tianjin, Guizhou, Inner Mongolia | Hainan |
| Uveitis with systemic diseases | Hainan, Ningxia, Guangdong | Guizhou, Sichuan, Tianjin, Beijing, Chongqing |
| Uveitis alone | Chongqing, Beijing,Tianjin, Sichuan,Guizhou | Ningxia, Hainan |
| Idiopathic uveitis | Tianjin, Beijing, Guizhou | Jiangxi, Hainan |
| Behçet’s disease | Ningxia, Guangxi | Beijing, Chongqing |
| Vogt-Koyanagi-Harada disease | Hainan, Anhui | Guizhou, Beijing, Tibet, Shaanxi, Shanghai, Tianjin, Ningxia, Chongqing |

**Table S1. The proportion of patients with different uveitis subtypes in Mainland China by provinces, 2006-2017**

| Table S2. Associations of uveitis onset with GDP per capita* | | | | | |
| --- | --- | --- | --- | --- | --- |
| Type | β%-Effect size (95% CI) | P Value | β%-Effect size (95% CI) | | P Value |
| Non-infectious uveitis | -1.92 (-2.61~-1.23) | <0.05 | -1.48 (-2.58~-0.39) | | <0.05 |
| Infectious uveitis | -0.24 (-3.67~2.97) | NS | -0.25 (-5.73~4.88) | | NS |
| Uveitis alone | -1.30 (-2.11~-0.51) | <0.05 | -1.17 (-2.49~0.13) | | NS |
| Uveitis with systemic diseases | -3.06 (-4.23~-1.86) | <0.05 | -1.96 (-3.84~-0.11) | | <0.05 |
| Idiopathic uveitis | -1.70 (-2.70~-0.74) | <0.05 | -1.10 (-2.69~0.46) | | NS |
| Behçet’s disease | -7.58 (-10.09~-5.17) | <0.05 | -6.32 (-9.95~-2.83) | | <0.05 |
| Vogt-Koyanagi-Harada disease | -1.048 (-2.88~-0.74) | NS | -0.29 (-2.51~2.99) | | NS |
| Overall | -1.85 (-2.52~-1.19) | <0.05 | -1.43 (-2.51~-0.37) | | <0.05 |
|  | no weights | | | population weights | |

*Poisson regression with generalized estimated equation (Poisson-GEE); CI= confidence intervals; NS=no significance

| Table S3. per capita GDP-mediated 2.5–uveitis relationship | | | | |
| --- | --- | --- | --- | --- |
|  | no climate controls | | climate controls | |
|  | β-Effect size (95% CI) | P Value | β-Effect size (95% CI) | P Value |
| below national per capita GDP | 0.011(0.0036~0.0185) | <0.01 | 0.0125(-0.0168~0.0417) | NS |
| above national per capita GDP | 0.0144(0.00819~0.0206) | <0.01 | 0.0124(0.0029~0.0220) | <0.01 |

CI= confidence intervals; NS=no significance
